# S100A14 serum level and its correlation with prognostic factors in breast cancer

**DOI:** 10.1186/s43046-020-00048-y

**Published:** 2020-09-28

**Authors:** Noor Al-Ashkar, Almoutassem Billah Zetoune

**Affiliations:** Department of Biochemistry and Microbiology, Faculty of Pharmacy, Damascus University, Damascus, Syria

**Keywords:** Breast cancer, S100A14, Serum, Tumor grade

## Abstract

**Background:**

Breast cancer is the most commonly occurring cancer in women worldwide. S100A14 is a novel important member of S100 proteins family. Its importance is due to its role in tumorigenesis and metastasis process. In this study, we aimed to determine serum levels of S100A14 protein in breast cancer patients and healthy individuals to know if it can be suggested as a new biomarker for breast cancer and to reveal whether it is correlated with cancer pathological features.

**Methods:**

This cross-sectional study was performed in two groups: study group contains 46 breast cancer patients (29 metastatic and 17 non-metastatic) and control group contains 22 healthy women. Enzyme-linked immunoabsorbent assay was performed to determine S100A14 protein levels in samples. Pathological data were obtained for each patient. The data were statistically analyzed using Kruskal-Wallis *H*, Mann-Whitney *U*, and Spearman correlation tests.

**Results:**

S100A14 serum levels were elevated in study group compared with control group (*P* < 0.05). S100A14 serum levels were significantly increased in distant breast cancer patients compared with regional breast cancer patients (*P* = 0.001). There was a strong positive correlation between serum S100A14 level and tumor grade (*r*_s_ = 0.713, *P* < 0.001).

**Conclusion:**

Our study indicated that S100A14 serum levels are elevated in breast cancer patients compared with control individuals. High S100A14 serum levels were correlated with poor tumor differentiation so it might have a prognostic significance for breast cancer tumors. The elevation of S100A14 levels in distant breast cancer patients suggests the ability of using serum S100A14 as a biomarker for detection of breast cancer metastasis.

## Background

S100 proteins constitute the largest subfamily of calcium binding protein of the EF-hand type. These low molecular weight acidic proteins play multifunctional roles as they have been reported to mediate a wide range of intracellular and extracellular functions [1]. While being inside the cell, S100 proteins perform different intracellular functions include calcium homeostasis, cell growth and survive, cytoskeletal arrangement, cell motility, and regulation of transcriptional factors [1]. On the other hand, extracellularly S100 proteins exert diverse cytokine-like functions as they are involved in transmission of signals in an autocrine/paracrine manner via interaction with a variety of cell-surface receptors such as the receptor for advanced glycation end products (RAGE) [1].

At least 25 members of S100 protein family have been discovered to date. S100A14 gene beside 22 other S100 genes are located at the chromosomal locus 1q21 which has been reported to be susceptible to genomic mutations in cancers, supporting that these proteins may be implicated in the progression of cancer and explaining the altered expression of most of S100 proteins in many cancers [1, 2].

S100A14, also known as BCMP84 (Breast Cancer Membrane Protein 84), was identified for the first time in 2002 while analyzing cell lines of human lung cancer and detected to have intracellular distribution [3].

The expression of S100A14 is considerably different in various tumors depending on the tissue that the tumor is arising from. S100A14 seems to be underexpressed in oral-gastrointestinal tumors, supporting that it may have a tumor suppressive functions in tissues rich in epithelial components, whereas cancers arising from tissues rich in mesenchymal components like breasts and ovaries showed overexpression of S100A14, supporting that this protein might have tumor-promoting functions in these tissues [2].

S100A14 has been reported to be overexpressed in breast cancer [4–7], ovarian cancer [8], and lung adenocarcinoma [9]. Furthermore, S100A14 has been revealed to promote cell invasion and metastasis in breast cancer [10, 11], lung adenocarcinoma [9], and hepatocellular carcinoma [12].

S100A14 high expression levels have been reported to correlate with poor breast cancer tumor differentiation, consequently it is suggested to be used as a prognostic factor for breast cancer patients [13]. It is also nominated to be a prognostic factor for lung adenocarcinoma patients [9], cervical cancer [14], and hepatocellular carcinoma patients [12].

An in vitro study on ovarian tumors supported the involvement of S100A14 in cell migration and invasion using a wound healing assay [15].

In this study, we detected the S100A14 serum levels in breast cancer patients and healthy individuals then we examined the association between S100A14 serum levels and cancer pathological features of these patients.

## Methods

This cross-sectional study included 68 women divided into two groups: control group consisting of 22 healthy women and study group consisting of 46 women (29 metastatic and 17 non-metastatic) who had been newly histologically diagnosed with primary breast cancer between September 2016 and April 2017. Both groups were age matched.

Study group samples were obtained prior to surgical treatment and before patients undergo any therapy. Patients with secondary cancers were excluded. Patients with missing required data in their medical records were excluded.

Patients’ clinical data were obtained from their medical records including histopathological subtype, TNM (tumor, nodes, and metastases) stages, hormones receptors status, Her2 receptor (human epidermal growth factor receptor 2) status, and tumor grade. These data were grouped according to the American Joint Committee on Cancer (AJCC) 2018 guidelines.

S100A14 protein serum levels were measured by enzyme-linked immunosorbent assay (ELISA) using commercial human kit from Sunred Biological Technology. ELISA assay was performed according to the manufacturer’s instructions.

The statistical study has been performed using IBM SPSS 23.0 software (SPSS Inc., Chicago, IL, USA). The data were analyzed using Kruskal-Wallis *H*, Mann-Whitney *U*, and Spearman correlation tests. Values were expressed as mean ± standard deviation (mean ± SD), while the results at *P* < 0.05 were considered significant.

## Results

### S100A14 levels in control group and cancer group

To verify the difference between S100A14 serum levels in the breast cancer group and the healthy control group, the serum concentration of S100A14 in the two groups was determined by ELISA.

**Fig. 1:**
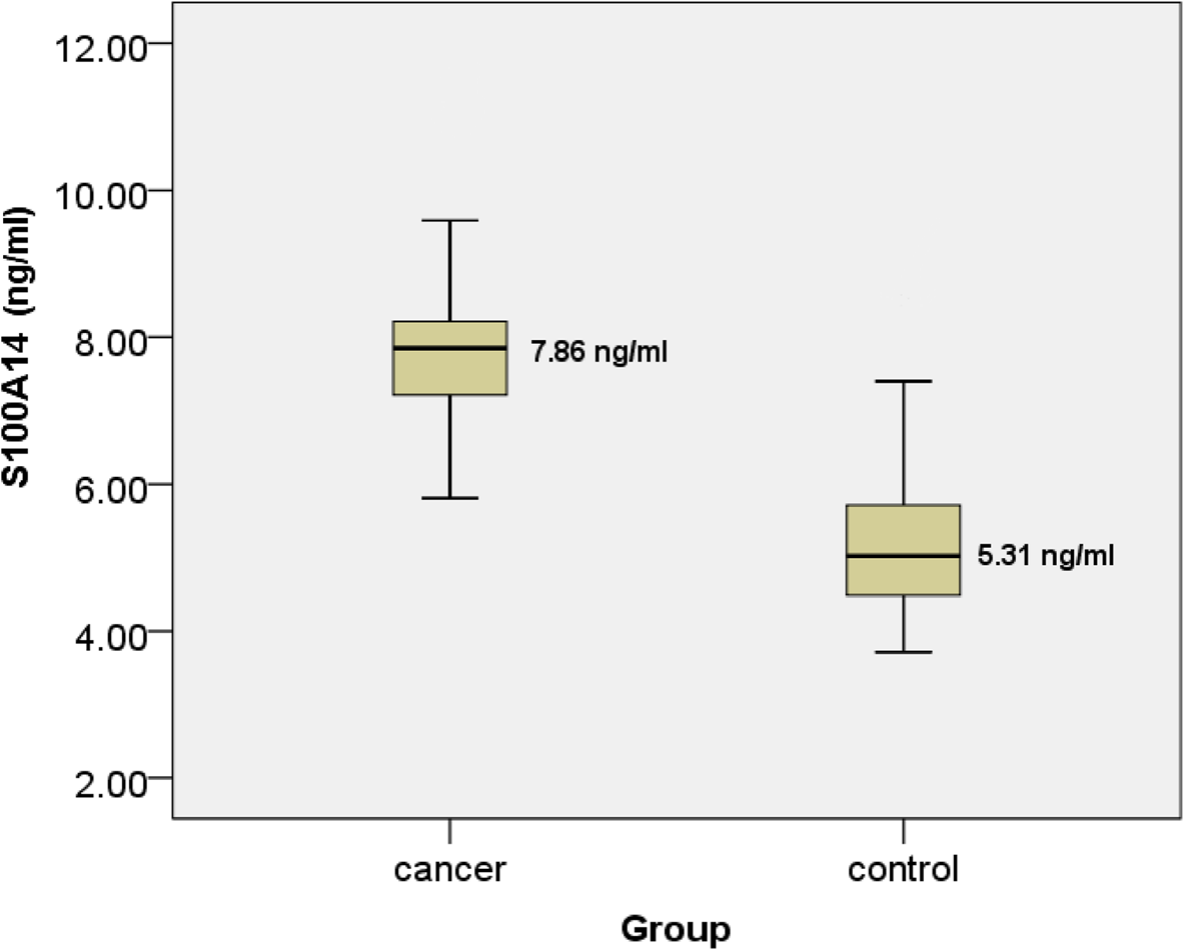
S100A14 serum levels in control group (*n* = 22) and cancer group (*n* = 46)

S100A14 serum levels were higher in breast cancer group (*n* = 46) compared with control group (*n* = 22). Statistically significant difference (*P* = 0.0001) was detected in S100A14 serum level in breast cancer group 7.86 ± 0.98 ng/ml (range 5.80–11.31 ng/ml) compared with control group 5.31 ± 1.19 ng/ml (range 3.72–8.53 ng/ml) as shown in Fig. 1.

### Comparison of S100A14 serum levels in breast cancer patients according to the cancer pathological features

The patients’ tests results were analyzed to investigate the association between S100A14 serum levels and the cancer pathological features of patients (Table 1).

**Table 1:** Comparison of S100A14 serum levels in patients according to the cancer pathological features

Cancer characteristics	Cases, ***n*** (%)	Serum S100A14 (ng/ml)	***P*** value*
**Tumor size (T)**			
1	18 (39.1)	8.22 ± 1.03	0.07
2	18 (39.1)	7.78 ± 0.93	
3	6 (13.4)	7.21 ± 1.06	
4	4 (8.7)	7.56 ± 0.29	
**Lymph node involvement (N)**			
0	7 (15.2)	8.48 ± 1.13	0.16
1	16 (34.8)	7.91 ± 1.23	
2	18 (39.1)	7.67 ± 0.74	
3	5 (10.9)	7.51 ± 0.29	
**Metastasis (M)**			
0	17 (37.0)	7.28 ± 0.67	**0.001**
1	29 (63.0)	8.20 ± 0.99	
**Histopathological subtype**			
IDC	34 (73.9)	8.01 ± 1.01	0.10
ILC	12 (26.1)	7.42 ± 0.82	
**Progesterone receptor (PR)**			
Positive	28 (60.9)	7.73 ± 0.98	0.34
Negative	18 (39.1)	8.07 ± 0.99	
**Estrogen receptor (ER)**			
Positive	24 (52.2)	7.68 ± 1.06	0.19
Negative	22 (47.8)	8.06 ± 0.88	
**Her2 receptor**			
Positive	30 (65.2)	7.76 ± 0.89	0.17
Negative	16 (34.8)	8.05 ± 1.17	

*Differences among T/N were analyzed by Kruskal-Wallis *H* test. Differences among M/histopathological subtype/PR/ER/Her2 were analyzed by Mann-Whitney *U* test. *P* > 0.05; *NS* not significant

No significant associations were found between S100A14 serum levels and tumor size, lymph node involvement, histopathological subtype, progesterone, and estrogen receptors status or Her2 receptor status. However, S100A14 serum levels in distant breast cancer patients were significantly higher than other breast cancer patients (*P* = 0.001).

### Correlation between S100A14 serum level and breast cancer tumor grade

Of 46 patients, 25 (54.3%) were with grade 2 tumors, while 10 (21.7%) and 11 (23.9%) were with grade 1 and 3, respectively. Spearman’s rank correlation analysis was used to analyze the correlation between S100A14 serum level and breast cancer tumor grade (Table 2).

**Table 2:** Correlation between S100A14 serum level and breast cancer tumor grade

Tumor grade	Cases, ***n*** (%)	Serum S100A14 (ng/ml)	Correlation coefficient^**α**^	***P*** value^**β**^
1	10 (21.74)	6.90 ± 0.64	0.713	**0.0001**
2	25 (54.35)	7.76 ± 0.40		
3	11 (23.91)	8.96 ± 1.17		

^α^The correlation coefficient was calculated by Spearman correlation analysis

^**β**^*P* > 0.05; *NS* not significant

**Fig. 2:**
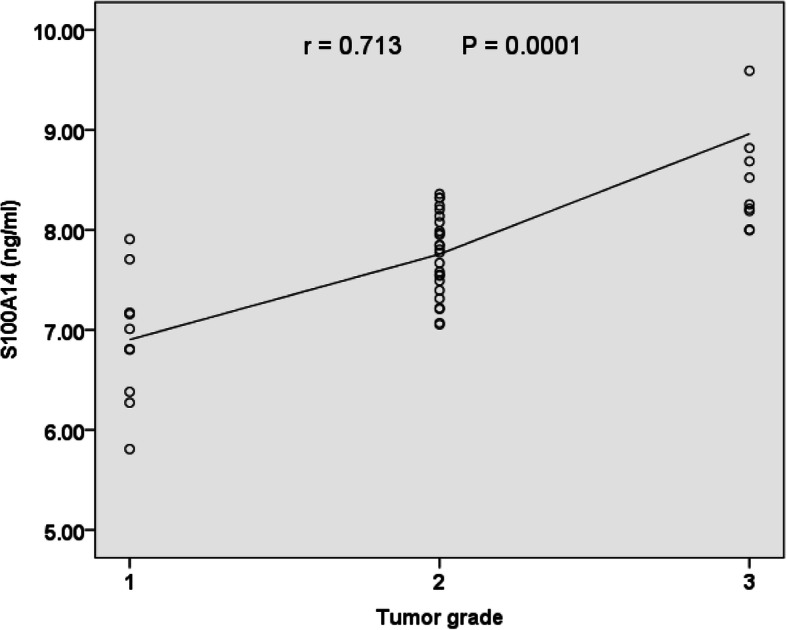
The correlation between S100A14 serum level and breast cancer tumor grade

There was a strong positive correlation between S100A14 serum level and tumor grade (*r*_s_ = 0.713, *P* = 0.0001) as shown in Fig. 2.

### ROC curve of serum S100A14 for discriminating between breast cancer group and healthy control group

**Fig. 3:**
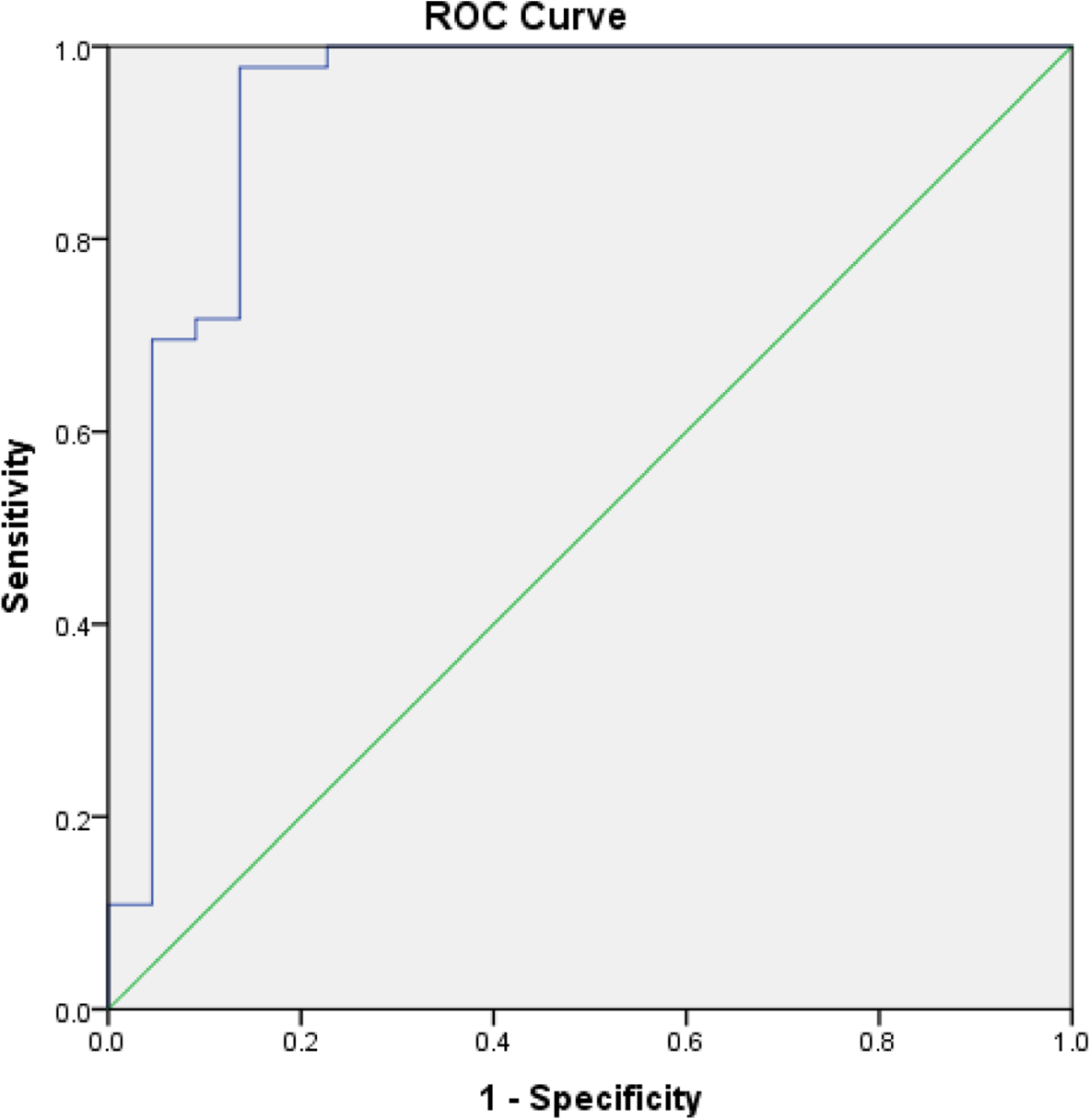
ROC curve of serum S100A14 for discriminating between breast cancer group and healthy control group

A receiver operating characteristic (ROC) curve with various cutoff levels of serum S100A14 was constructed to study the ability of this protein to predict breast cancer (Fig. 3) and area under the curve was calculated (Table 3).

**Table 3:** Area under the curve description

Area under the curve	Standard deviation	***P*** value*
0.931	0.043	0.0001

*P > 0.05; *NS* not significant

At the cutoff point 6.21 ng/ml, 93.8% of breast cancer patients were above this point, whereas 95% of healthy control individuals were below this point. The sensitivity and specificity at this point were 97.8% and 86.4%, respectively.

## Discussion

Breast cancer is the most frequent cancer among women accounting for 24.2% of all female cancers and it is the most common cause of death from cancer in women as its mortality ratio is about 15% [16]. Distant breast cancer causes the majority of these deaths because cancer in these cases has reached organs outside of the breasts like bones, lungs, liver, or brain. Although breast cancer treatment methods are increased and improved, the 5-year survival rate of distant breast cancer patients remains relatively low as the distant metastases may cause a secondary cancer in other organ. According to the American Cancer Society, the 5-year relative survival rate for distant breast cancer is 27% ,whereas it is 86% for regional breast cancer [17].

The early detection of breast cancer and its metastasis opens the door to more treatment options, higher treatment efficacy and higher survival rates. It is also important for improving patient prognosis and outcomes as it helps saving their lives. At present, the detection of distant metastases has limitations and requires long time and high cost. The development of additional molecular biomarkers is strongly required and a simple non-invasive test on an easy obtainable sample for the early detection of patients with breast cancer metastasis is therefore sought after.

S100A14 is one of the newest members of the S100 calcium binding protein family that has gained increased interest in cancer researches as it plays various roles that are related to carcinogenesis such as cell proliferation, cell invasion and motility, cell differentiation, and regulation of transcription factors like tumor protein p53 [1]. Many studies revealed that altering in S100A14 expression is associated with cancer progression and prognosis [10, 18, 19]. Previous studies have also clearly revealed the presence of S100A14 in serum as it is secreted by cancer cells and exerts extracellular functions [20, 21]. Therefore, we aimed in this study to determine the levels of S100A14 protein in serum samples of breast cancer patients and healthy individuals.

Studies suggested many theories about the working mechanism of S100A14. A study on esophageal squamous cell carcinoma reported that the extracellular S100A14 protein binds to RAGE receptor (receptor for advanced glycation end products) leading to modulating of RAGE signaling, as the engagement of RAGE with S100A14 triggers activation of mitogen activated protein (MAP) kinase and nuclear factor κB (NF-κB) signaling pathways [2]. While the over signaling of MAP kinase pathway causes altering in cell proliferation and survival to an abnormal manner, NF-κB-signaling pathway establishes a microenvironment which is crucial for either cancer initiation or development, or both by increasing cellular metabolic activity and cell division [22].

Another study identified CCL2 (chemokine (C-C motif) ligand 2) as a downstream target of S100A14. CCL2 is a chemokine secreted from cancer cells, it initiates chemokine cascade that results in TAM (tumor-associated macrophage) recruitment [10]. These TAMs contribute to cancer progression by secreting extracellular vesicles contain signal molecules like PDGF (platelet-derived growth factor) that promote tumor growth, VEGF (vascular endothelial growth factor) that induce angiogenesis, and MMPs (matrix metalloproteinases) which are important for tumor migration, invasion, and metastasis [23].

These findings may explain the elevation in serum levels of S100A14 protein in breast cancer patients compared with healthy individuals in our study. Moreover, our results agree with a recent study suggested that the S100A14 serum level may be used as a potential biomarker for detecting breast cancer [10].

Our study revealed that S100A14 serum levels in distant breast cancer patients were significantly higher compared with other breast cancer patients. This result suggests that S100A14 may be implicated in cancer metastasis process.

This result supports another study that demonstrated that S100A14 can promote breast cancer cell invasion by regulating MMP2 (matrix metalloproteinase2) transcription in a p53-dependent way, as MMP2 is a protease responsible for degrading the multiple components of the extracellular matrix of cancer cell which is an important initial step in cancer cells invasion and metastasis process [11].

Furthermore, our study suggests that S100A14 serum level may be used as a biomarker that enhances our accuracy in identifying patients with distant metastasis, and hence provide useful information for medical management.

Strong correlation was found in our study between S100A14 serum level and the tumor grade. The higher the level of serum S100A14, the poorer the differentiation of the tumor, which indicates that this protein might inhibit cell differentiation. This result agrees with a previous report that demonstrated that high S100A14 expression was correlated with poor tumor differentiation. Moreover, that study suggested that S100A14 overexpression might be a significantly prognostic indicator of patients with breast cancer [13].

This study has many limitations which were because of limited self-funding; the first limitation is the number of participants which we could not make bigger. The second limitation is the lack of an in vitro or histological supporting study.

## Conclusion

Our study has revealed that S100A14 serum levels were significantly higher in breast cancer group compared with control group. Large-scale studies are required to confirm these results and to study if it can be used for the detection of breast cancer as a screening test.

Our study has demonstrated that S100A14 serum levels in distant breast cancer patients were significantly higher compared with other breast cancer patients.

A strong correlation has been found between S100A14 serum level and the tumor grade in our study, which indicates that this protein might inhibit cell differentiation and may be a potential prognostic biomarker for breast cancer patients.

This study revealed that S100A14 serum level of 6.21 ng/ml is nominated to be a good cutoff point to distinguish breast cancer patients from healthy individuals.

More studies with larger sample sizes should be carried out to support these findings.

## Data Availability

All materials and all data generated during this study are included in this research article.

## Abbreviations

ELISA: Enzyme-linked immunoabsorbent assay
RAGE: Receptor for advanced glycation end products
BCMP84: Breast Cancer Membrane Protein 84
TNM: Tumor, nodes and metastases
Her2 receptor: Human epidermal growth factor receptor 2
SD: Standard deviation
PR: Progesterone
ER: Estrogen
MAP: Mitogen activated protein
NF-κB: Nuclear factor κB
CCL2: Chemokine (C-C motif) ligand 2
TAM: Tumor-associated macrophage
PDGF: Platelet-derived growth factor
VEGF: Vascular endothelial growth factor
MMPs: Matrix metalloproteinases
MMP2: Matrix metalloproteinase2
ROC: Receiver operating characteristic
